# Practice of opportunistic cervical cancer screening and health education among health workers in Ogun State, Nigeria: A qualitative study of barriers and facilitators

**DOI:** 10.1371/journal.pone.0316883

**Published:** 2025-09-11

**Authors:** Tope Olubodun, Abimbola Olaniran, Ibraheem Olayemi Awowole, Ephraim Ohazurike, Tolulope Soyannwo, Olusegun I. Adebisi, Oludolamu Oluyemisi Adedayo, Kamarudeen Olaitan Issa, Solomon Olorunsaiye Olorunfemi, Omotola Omowunmi Akinmolayan, Olumide Ayodeji Isarinde, Ibrahim Oladimeji Boladale, Gbemiga Adekunle Ajewole, Imran O. Morhason-Bello

**Affiliations:** 1 Department of Community Medicine and Primary Care, Federal Medical Center, Abeokuta, Ogun State, Nigeria; 2 KIT Royal Tropical Institute, Amsterdam, Netherlands; 3 Department of Obstetrics and Gynaecology, College of Medicine, Obafemi Awolowo University, Ile Ife, Osun State, Nigeria; 4 Department of Obstetrics and Gynaecology, College of Medicine, University of Lagos, Lagos, Lagos State, Nigeria; 5 Department of Obstetrics and Gynaecology, College of Medicine, University of Ibadan, Ibadan, Oyo State, Nigeria; University of Jos Faculty of Medical Sciences, NIGERIA

## Abstract

**Background:**

The burden of cervical cancer is highest in low- and middle-income countries. In Nigeria, where organized cervical cancer screening programs are lacking, opportunistic screening during maternal healthcare visits may enhance screening uptake. This study is aimed at understanding the practice of opportunistic cervical cancer screening and health education, and the barriers and facilitators experienced by health workers practicing in antenatal and postnatal clinics in Ogun State, Nigeria.

**Methods:**

This is a qualitative cross-sectional study. In-depth interviews were conducted among 43 health workers – doctors, nurses and community health extension workers, working in antenatal and postnatal clinics in public primary, secondary and tertiary health facilities selected by quota sampling. A hybrid thematic data analysis approach, combining deductive and inductive methods was employed.

**Results:**

Health education on cervical cancer prevention was not done in most health facilities. Where cervical cancer health education was practiced, it was done mostly prior to family planning provision, and sometimes at antenatal, postnatal/infant immunization clinics. Facilities for cervical cancer screening was not available in most of the health facilities and patients had to travel long distances to tertiary facilities to have a Pap smear done. Barriers to cervical cancer screening and health education include, high cost of screening, manpower shortage, fear of positive result among patients, poor awareness among patients, and religious and cultural beliefs. The major facilitators to screening and health education mentioned were passion for their work and the desire that no woman should die from preventable cancers.

**Conclusion:**

To address identified barriers, the government should enhance health worker training, ensure adequate staffing, improve the availability of screening equipment and reagents and make screening free/affordable. Community mobilization efforts should be intensified to increase awareness and promote accessibility. Integrating cervical cancer health education and screening into routine antenatal and postnatal care is essential for improving uptake.

## Introduction

Globally, cervical cancer kills more than 300,000 women annually and is the fourth most common cancer worldwide [1,2]. There were 604,000 new cases of cervical cancer globally in 2021 [1]. Cervical cancer disparities exist with the disease being more prevalent in low- and middle-income countries due to inadequate screening and treatment [3] In high income countries, age standardised incidence rate (ASIR) of cervical cancer is 9.6 per 100 000 women compared to 26.7 per 100 000 women in low-income countries, where late presentation and high mortality is prevalent [4].

Cervical cancer can be prevented by vaccination against the human papilloma virus (HPV), and adequate screening and treatment of precancerous lesions [5]. The Global Strategy for Cervical Cancer Elimination adopted in 2020 by the World Health Assembly states that all countries should attain and maintain an ASIR of 4 per 100 000 women in order to eliminate cervical cancer [6]. To achieve this, ninety percent of girls should receive HPV vaccination by the age of 15 years, 70% of women should be screened with a high-performance test by the age of 35 years and again by age 45, and 90% of women with precancerous lesions and cervical cancers should be treated [6]. ASIR of cervical cancer in Nigeria however is 18.4 per 100 000 [7] and uptake of cervical cancer screening is still very low [8–13] with some studies reporting as low as zero uptake [8,14] and one percent uptake [9,15]

Nigeria is yet to adopt an organised screening programme, although the National Cancer Control Plan 2018–2022 had a target to achieve greater than 50% screening of eligible population for common cancers by 2022 [16]. Mostly, screening for cervical cancer in Nigeria is opportunistic, when a patient visits a health professional or when symptoms warrant cervical cancer screening. Some civil society organisations, non-Governmental organizations and State Governments infrequently conduct cervical cancer screening in communities using visual inspection with acetic acid/Lugol’s iodine (VIA/VILI). While we await organised cervical cancer screening programmes in Nigeria, opportunistic screening should be optimised in the meantime.

Nigeria has a large population of women of reproductive age, of about 40 million women [17], and with Nigeria’s fertility rate being as high as 5, maternal health services serve as a window of opportunity for women to receive health education on cervical cancer and get screened for the disease [18]. At antenatal and postnatal clinics, women can be educated on cervical cancer prevention. And at postnatal visits, and subsequent visits, they can be screened for the disease or referred to screening centers.

Cervical cancer screening at the sixth-week postnatal visit is a recommended practice in many settings [19]. Health education is usually being given at antenatal and postnatal clinic visits, on a variety of topics that affect maternal and child health in Nigeria [20,21]. This period of opportunity can also be used to provide information on cervical cancer prevention. It is however, not well understood if this is the case, due to paucity of research on this subject. This qualitative study aimed at understanding the practice of opportunistic cervical cancer screening and health education, and the barriers and facilitators experienced by health workers practicing in antenatal and postnatal clinics in Ogun State, Nigeria. Findings from the study unravel the present practices in secondary cervical cancer prevention in health facilities in Ogun State, which can inform control efforts of cervical cancer in the country.

## Methods

### Study setting

Ogun State is located in Southwest Nigeria and has a projected population of 6,379,500 in 2021 [22]. Ogun State has 3 senatorial districts and 20 Local Government Areas (LGAs). The State has urban cities and rural settlements, and the state capital is Abeokuta [23]. There are 3 public tertiary hospitals – two of which are multi-specialist hospitals and the third, a neuropsychiatric hospital. There are 29 public secondary hospitals and 500 primary health centers (PHCs) in the State. There were 895 registered and functional private health facilities in the State, as at June 2024.

### Study population and study design

This study on cervical cancer opportunistic screening was part of a qualitative research project that aimed to assess the practice, facilitators and barriers of health workers towards breast and cervical cancer opportunistic screening in Ogun State, Nigeria. This research is a qualitative cross-sectional study that utilised in-depth interviews (IDIs). The study population were health workers (e.g., doctors, nurses, community health extension workers) working in antenatal and postnatal clinics in public primary, secondary and tertiary health facilities in Ogun State, Nigeria. Health workers who have been working in the selected health facility for at least one year were eligible for the study.

### Sampling

Health workers were selected from primary, secondary and tertiary health facilities that provide maternal health services – antenatal, delivery and postnatal services. Quota sampling was used to select the health facilities. There are three senatorial districts in Ogun State. Eleven health facilities, 10 health facilities and 6 health facilities were selected from Ogun Central, Ogun East and Ogun West senatorial districts. The number of health facilities selected from each senatorial district was proportionate to the total number of public health facilities available in each district, ensuring adequate representation of both urban and rural settings. Health workers who have contact with pregnant women and mothers during antenatal and postnatal visits, either through consultations or health education/health talk sessions and had been working in that health facility for at least one year, were selected purposively. In tertiary and secondary health facilities, one doctor and one nurse each were selected. In primary health centers, one nurse/CHEW was selected.

### Data collection

Data was collected from December 3^rd^ 2023 to January 15^th^ 2024. The selected participants were contacted, and a mutually convenient date and time was chosen for each IDI. Forty-three health workers were interviewed. The interviews were guided by a topic guide that contained open-ended questions.

Six resident doctors from the public health department of one of the tertiary hospitals in the state were trained in qualitative data collection and they subsequently served as the research officers for the study, assisting in data collection. The data collectors underwent a two-day training workshop on qualitative interviewing techniques, probing strategies, and ethical considerations to ensure consistency in data collection. The data collectors asked questions, guided by the topic guide. The topic guide covered themes such as health workers’ knowledge of cervical cancer and screening, practices related to cervical cancer screening and education, availability of screening facilities, perceived barriers and facilitators and recommendations for cervical cancer screening and education. The topic guide also had similar questions related to breast cancer screening as the research project was on breast and cervical cancer opportunistic screening. The questions were followed by prompts and probes to get deeper understanding of the subject. Each interview lasted about one hour. The interviews were audio-recorded, and the data collector also took notes, to supplement the recording and capture non-verbal cues.

### Data management and analysis

The audio recordings were transcribed verbatim. Data analysis was done with the aid of NVivo 13 qualitative software. A hybrid thematic analysis approach was employed, combining both deductive and inductive methods. Initially, a deductive coding framework was applied, based on a priori themes derived from the interview topic guide. Relevant sections of the transcripts were systematically mapped to these predefined themes, and subthemes were developed as needed. In addition, an inductive approach was used to identify emerging themes from the data that did not align with the initial thematic framework. These newly identified codes and themes were developed iteratively through close reading of the transcripts, allowing for a more comprehensive understanding of the data.

### Ethical approval

Ethical approval was obtained from the Ogun State Health Research and Ethics Committee (OGHREC/467/161). Verbal approval was obtained from facility management and recorded in official study logs. Written informed consent was obtained from the participants prior to the interviews. Confidentiality was maintained by utilising unique identifiers during data collection, data analysis and report writing. Transcripts were securely stored in password-protected files, accessible only to authorized researchers. All procedures were carried out according to the declaration of Helsinki.

## Results

### Background characteristics of participants

The mean age of participants was 43.4 ± 1.4 years and most participants 29 (67.4%) were females. Fourteen (32.5%) of the participants were practising in primary health centers, 24 (55.8%) in secondary health facilities and 5 (11.6%) in tertiary health facilities. Twenty-five (58.1%) were nurses, 15 (34.9%) doctors and 3 (7%) were CHEWS. Majority, 29 (67.4%) were senior cadre health workers, majority 35 (81.4%) had more than 20 years’ experience as health workers, and most, 29 (67.4%) had been in their current place of assignment for 2–5years. (Table 1)

**Table 1:** Background characteristics of participants.

Variable	Frequency (n = 43)	Percentage
**Age (years)**		
20-29	2	4.7
30-39	10	23.3
40-49	20	46.5
50-59	11	25.6
Mean ± SD	43.4 ± 1.4	
**Sex**		
Female	29	67.4
Male	14	32.6
**Level of health facility**		
Primary	14	32.6
Secondary	24	55.8
Tertiary	5	11.6
**Occupation**		
CHEW	3	7
Nurse	25	58.1
Doctor	15	34.9
**Cadre**		
Junior cadre	8	18.6
Middle cadre	6	14
Senior cadre	29	67.4
**Years of experience**		
2-9 years	8	18.6
≥ 20 years	35	81.4
**Years on job**		
2-5 years	29	67.4
6-10 years	6	14
>10 years	8	18.6
**Senatorial district of practice**		
Ogun Central	18	41.9
Ogun East	16	37.2
Ogun West	9	20.9
**Place of practice**		
Urban LGA	22	51.2
Rural LGA	21	48.8

The themes and sub-themes from data analysis are shown in Table 2.

**Table 2:** Themes, sub-themes and categories from data analysis.

	Themes	Sub-themes	Categories
1	**Knowledge of cervical cancer screening**		
2	**Cervical cancer health education**	Practice of health education in various health facilities Practice of cervical cancer prevention health education Barriers to cervical cancer health education Facilitators of cervical cancer health education	Situations when cervical cancer health education is practiced Contents of cervical cancer health education
3	**Cervical cancer screening**	Availability of facilities for cervical cancer screening Practice of cervical cancer screening Barriers to cervical cancer screening faced by health workers Barriers to cervical cancer screening faced by patients Facilitators of cervical cancer screening	
4	**Recommendations for cervical cancer health education and opportunistic screening**		

### Knowledge of cervical cancer screening

Most of the health workers knew about cervical cancer screening and could provide explanations on what cervical cancer screening means. Health workers said that screening helps detect cancer early, prevents its spread and is done for sexually active women. However, there were some gaps in the types of screening methods known as some health workers could not mention names of screening tests and only a few mentioned HPV testing. When health workers were asked the question; Can you tell me what you know about cervical cancer screening, 73.3% of doctors mentioned Pap smear, and 60.6% of nurses mentioned VIA. Even though they provided some explanation on what cervical cancer is, 66.0% of CHEWS did not mention any cervical cancer screening method and also 40.0% of nurses. Fifty percent of health workers in secondary health facilities mentioned Pap smear and all health workers in tertiary health facilities mentioned Pap smear while only 28.6% of primary health workers mentioned Pap smear [Table 3]. Only three respondents mentioned HPV testing as a cervical cancer screening method, of which all were doctors (1 secondary and 2 tertiary). Some health workers also mixed up the names of screening tests, providing description of a particular screening method and giving it another name.

**Table 3:** Methods of cervical cancer screening mentioned by health workers.

Variables	Type of cervical cancer screening method mentioned				Total number of health workers
	VIA	Pap test	HPV testing	Did not mention any method	
**Type of health worker**					
Doctors	4(26.6%)	11(73.3%)	2(20.0%)	2(13.3%)	15
Nurses	15(60.0%)	9(36.0%)	0(0%)	10(40.0%)	25
CHEWS	0(0%)	1(33.3%)	0(0%)	2(66.6%)	3
**Level of health facility**					
Primary	5(35.7%)	4(28.6%)	0(0%)	6(42.9%)	14
Secondary	5(20.8%)	12(50.0%)	1(4.2%)	8(33.3%)	24
Tertiary	5(100.0%)	5(100.0%)	2(40.0%)	0(0%)	5

*“I don’t know the name. They use a reagent. And they apply it in the cervical wall... I think they usually say, they expect a kind of changes. Yes, a kind of changes that appear, I think they call it Pap smear or something.” [Secondary hospital nurse; Principal Nursing Officer; Female; 32 years old]*

A few health workers equated cervical cancer screening to just inspecting the cervix during vaginal examination, without the use of acetic acid/Lugol’s iodine. Some participants felt screening was only done to detect cancer of the cervix in women and did not mention that screening also helps detect pre-invasive lesions.

*“Uhm.. cervical cancer screening is also done to detect if a person has a potential of having cervical cancer. If a person has …been infected by HPV, human, human papilloma virus. It is done mainly by using acetic acid. Yeah, by using acetic acid.” [PHC nurse; Nursing Officer; Female; 24 years old]*

*“Well cervical cancer screening is... I think is Pap smear. Usually, if patient is symptomatic, or if patient is sexually exposed, you want to do cervical screening... But basically Pap smear. Though we don’t do it in this facility.” [Secondary hospital doctor; Medical officer; Male; 34 years old]*

*“Cervical cancer screening... That is uhm…. When you subject your patient to test in the cervix, to detect if there’s any cancerous cells in the cervix... You know... laboratory tests just to know if there is any growth of cancer cells in the cervix.” [Secondary hospital nurse, Chief Nursing Officer; Female; 52 years old]*

*“Emmm.. Cervical cancer screening…, we all know the gold standard is the Pap smear. But we don’t do the Pap smear here. What we do here basically is the ehmm, the clinical ehm inspection examination.” [Secondary hospital doctor; Senior Medical Officer; Male; 42 years old]*

*So, the aim of cervical cancer screening is to detect the early pre-malignant phase and the early phase of malignancy regarding cancer of the cervix…. The advantage of the screening is to pick some of these malignancies at a very very early stage, where it can either be treated by local surgery, excision, cryotherapy or cold knife coagulation or diathermy or hysterectomy…” [Tertiary hospital doctor; Consultant; Male; 42 years old]*

### Cervical cancer health education

#### Practice of health education in various health facilities.

Health education is practiced in all the health facilities during antenatal visits, in the form of group health education. The tertiary health facilities have a separate day, each week for postnatal clinics (6 weeks after delivery), and usually carry out group health education on those days. Most secondary health facilities and PHCs don’t have a day of the week set aside for postnatal clinics and some only attend to postnatal mothers when they come for the six weeks immunization of their babies, and they have complaints. Thus, the infant immunization clinics, can double as a form of postnatal clinic. However, group health education is not routinely done in some postnatal/infant immunization clinics. In most health facilities, group health education is usually conducted by nurses.

Health education topics during antenatal visits include breastfeeding and exclusive breastfeeding, maternal nutrition, personal hygiene, environmental hygiene, breast self-examination, warning signs in pregnancy, signs of labour, and importance of good health seeking behaviour.

*“We have health talk every antenatal clinic and we make sure to touch every aspect of health that is necessary for a pregnant woman. And we have a schedule which we follow so that we don’t miss anything. And we make sure that everybody that is in attendance listens and participates in the health talk.” [PHC Nurse; Nursing Officer; Female; 24 years old]*

#### Practice of cervical cancer prevention health education.

Many health workers seldom counsel or educate women on cervical cancer prevention. Some said they educate women more on breast cancer, than cervical cancer prevention. However, some health workers said that, with the introduction of HPV vaccination, they have also included cervical cancer screening as well as HPV vaccination in their health talks.

*“We concentrate most on breast cancer…” [Secondary hospital nurse; Assistant Chief Nursing Officer; Female; 49 years old]*

*“Hardly, hardly… Cervical cancer doesn’t usually come up...Hardly” [Secondary hospital doctor; Principal Medical Officer; 47 years old]]*

*“During our health talk for antenatal, breast cancer is part of our topics that we are talking about. But for cervical screening, we don’t really stress it. But when the vaccine came out - this vaccine they gave to us - So, since that time, we’ve included cervical screening into our health talk”. [PHC nurse; Assistant Chief Nursing Officer; Female; 44 years old]*

#### Situations when cervical cancer health education is practiced.

Cervical cancer health education is sometimes done for women seeking family planning, sometimes during antenatal visits, postnatal clinics or immunization clinics. In some centers, education about the prevention of cervical cancer is only done at gynaecology clinics. Some health workers, mostly doctors said that they only discuss cervical cancer screening when patients present with suggestive symptoms.

*“Yes, we do, especially during the family planning session. That is when we will normally tell them about cervical health education. …. When we are carrying out some procedure like IUD, we normally tell them”. [PHC nurse; Principal Nursing Officer; Male; 48 years old]*

*“We do it in my facility, … It’s mostly done during antenatal, post-natal and whenever they come to the family planning clinic. We make them to understand - in case they notice any changes maybe in their private part or discharges, that they should come to the hospital and lodge complaints.” [Secondary hospital nurse; Principal Nursing Officer; Female; 42 years]*

*“Yeah, as I said before, emm, one of our hallmarks of postnatal clinic is - especially when we are discharging them from the clinic - is that, as we are educating them about family planning, we educate them also about cervical cancer screening... for them to join the program (car honking) and get screened regularly.” [Tertiary hospital doctor; Senior resident doctor; Male; 41 years old]*

*“Cervical cancer education is usually not discussed in antenatal clinic visits. But most times, during our gynae clinic visits is when we take time to talk to women about cervical cancer screening. … Even if they come to the clinic for benign cases, we still take our time to send them for pap smear and other cervical cancer screening, just as an opportunity to screen them but we don’t discuss cervical cancer in antenatal clinic.” [Tertiary hospital doctor; Consultant Obstetrician & Gynaecologist; Male; 42 years old]*

*“It depends on where we see cases like that........We don’t routinely do it. It is when we see cases that are related, we will discuss with them.” [Secondary hospital doctor; Senior medical officer; Male; 40 years old]*

#### Contents of cervical cancer health education.

Regarding health education on the prevention of cervical cancer, some health workers majorly advice their clients on avoiding risk factors like multiple sexual partners and multiple pregnancies. Health education also focused on telling patients to present in the hospital when they have symptoms suggestive of cervical cancer such as post-coital bleeding. A few health workers actually counsel on screening, when symptoms warrant it. Many health workers had incorrect knowledge of cervical cancer risk factors and they passed this wrong information to women during health education. For example, some health workers felt douching and use of inappropriate sexual activity lubricants like saliva, was a risk factor for cervical cancer.

*“We actually emphasize on sexual partners…”. [Secondary hospital doctor; Senior Medical Officer; Female; 35 years old]*

*“We educate on personal hygiene. We advise them to have one partner, not to be moving from one partner to another. We try to explain the mode of transmission to them, how it can be treated…. We do tell them especially during antenatal clinic, that they should not deliver much children; that they can be putting pressure on their uterus and the cervix.” [PHC nurse; Chief Nursing Officer; Female; 58 years old]*

*“We don’t normally tell them on the screening, but we tell them on the prevention. Stop using... maybe something like ehm… harmful ingredients to wash the vagina. We normally tell them all this things are harmful. We let them to know that they are harmful, and it can cause a lot of diseases in the future.” [Secondary hospital nurse; Assistant Chief Nursing Officer; Female; 49 years old]*

*“Sure, we do. We do give them health talks, both on cervical and the breast cancer. We do teach them to prevent using soap and washing their vagina - That is not good for them. And then, you know - douching. And they should change their pad often and often.” [Secondary hospital nurse; Principal Nursing Officer; Female; 43 years old]*

*“Yes, the nurses do. Yes, they do, they discuss with them about signs to see - If there is any excessive bleeding. If there is any bleeding, they should make sure they come to the hospital.” [Secondary hospital doctor; Senior Medical Officer; Female; 34 years old]*

*“Ok! So, when I have one- on- one encounter with them, I try to tell them... There are some people, they will say that their husband want to use saliva as lubricant. I try to discourage that as much as possible, that they should get lubricants like KY gel. It makes it easier. …. They should avoid using saliva as lubricant or any form of oral sex.” [PHC nurse; Nursing Officer; Female; 24 years old]*

*“We don’t really talk about much on cervical cancer, but when we have patients that have infection, we do speak on cervical screening... When last did you do your cervical examination screening?” [PHC nurse; Assistant Chief Nursing Officer; Female; 44 years old]*

#### Barriers to cervical cancer health education.

Many health workers said they don’t have any challenges with giving health education, as it only entails them talking to the patients. Other health workers mentioned manpower shortage and excess workload, leading to time constraints in giving health education. Language barrier was mentioned by only a few, as most of the health workers could speak the local language. Also, in a few cases, when there were clients who neither understood English or the local language well enough, language became a barrier. Another barrier experienced sometimes was poor audience from the patients as they are sometimes in a haste to leave the health facilities back to their work/businesses.

Lack of training on health education was also mentioned as a barrier to cervical cancer health education. A male doctor mentioned gender differences as a challenge to health education as women confide better and feel freer with female health workers.

*“We don’t have any challenge in offering health education. Because someone who cannot speak English, you speak to them in their mother tongue.” [Secondary hospital doctor; Principal Medical Officer; Male; 53 years old]*

*“In this environment, gender differences are there. The females feel freer with their female counterparts. They open up more to the female counterparts than you, a male. Despite the fact that you are a doctor and will help them out.” [Secondary hospital doctor; Principal Medical Officer; Male; 45 years old]*

*“It is still shortage of manpower. Because for two nurses to be manning the ward and still come back to give health education... Then also,- the time. Because for you to give a quality health education, you need to have enough time…. When the workload is too much, you won’t be free with your patients.” [Secondary hospital nurse; Principal Nursing Officer; Female; 36 years old]*

*“Sometimes we have people that do not understand the lingua franca, English or Yoruba. So, you need a translator. In short, we have a whole lot of them, because this community is mixed. We have Fulani’s. We have people from Cotonou. We have Igedes. We have a lot of people. So most of the things I might want to tell them gets lost in translation.” [Secondary hospital doctor; Principal Medical Officer; Male; 47 years old]*

*“They all want to go back to their workplace. Nobody is ready to listen. They feel we are talking too much. It’s like they don’t really see… They don’t really attach importance to health education perse. They are always in a hurry to go back to their workplace... And also, the health workers sometimes. Let me say - we don’t really prepare ahead for their questions.” [PHC nurse; Nursing Officer; Female;24 years old]*

#### Facilitators of cervical cancer health education.

Many health workers said the desire that no woman should suffer from preventable cancers was what encouraged them to offer cervical cancer health education. Some health workers mentioned that patients’ interest and inquisitiveness to learn encouraged them to provide more information. Positive response from the patients, e.g., carrying out the preventive measures like screening, was mentioned by another health worker as a motivating factor. High educational status of the clients was mentioned as a factor which makes counselling easier as the client can easily understand what is being taught. One health worker explained that providing health education to pregnant women was the norm, and that in itself is a facilitator of breast and cervical cancer health education.

*“I think it is based on what we are hearing nowadays - of people dying of cervical cancer, of cancer of different types. So, we always want to guide our patients... You know, against having such.” [Secondary hospital nurse; Chief Nursing Officer; Female;52 years old]*

*“In all sincerity, the motivation comes from the clients. When you have a client who you are sure is interested, then you really want to help the client. But when the patient is interested in something else, then when you’re not overburden, then you can help. But when you have so much to do, you get discouraged.” [Secondary hospital doctor; Principal Medical Officer; Male; 44 years old]*

*“It depends. Most of the time, the educational level or the status of the patient matters… If you are explaining to a pepper seller that there is a disease called cancer, they will not understand. They will not relate as well, compared to somebody that is more enlightened - that is probably exposed, and has heard things like that. Educational status makes your job easy, which means you do less counselling.” [Secondary hospital doctor; Senior Medical Officer; Male; 42 years old]*

*“You know, we need to update our knowledge. If we have the latest information about these services, we’ll be able to provide it and let our people know that this is the latest information. You know, the medicine is changing every time.” [PHC nurse; Principal Nursing Officer; Male; 48 years old]*

*“There is nothing stopping me from giving education. My own is, I will tell them. I will give the advice... Some people, they are ready to listen to you, while some people, they will tell you they will never have the disease…and which we don’t pray so.” [PHC CHEW; Female; 45 years old]*

### Cervical cancer screening

#### Availability of facilities for cervical cancer screening.

Most participants said facilities for cervical cancer screening were not available nor geographically accessible to their clients. Some added that they had to refer clients to tertiary centers which were distant from their own facilities. Some participants stated that they had facilities for cervical cancer screening in the past, but it was no longer available. In both tertiary centers however, facilities for screening with Pap smear are available. A secondary facility had facilities for VIA which is used for screening of HIV patients, made available by partnership with an international NGO. HPV testing is not available in any of the health facilities.

*“Well, … this facility is situated in a local area. And, you know, it’s a surbub. It’s in the bush...(laughter)... So, most of the things we do here, we improvise. So, when it comes to major things like this screening and all that, we do send them to town. Ijebu Ode is our town here.” [Secondary hospital doctor; Principal Medical Officer; Female; 45 years old]*

*“There is no facility for it, there is no equipment and no medical personnel that are specialty for it. We don’t have. So that’s why it’s not available here.” [Secondary hospital nurse; Principal Nursing Officer; Female; 41 years old]*

*“Okay, cervical cancer screening? Yes. Some years back, we do it here. We were using acetic acid to check the cervix so as to detect anybody with this papilloma. But for years back, we could not get acetic acid. So, we stopped doing the tests here.” [Secondary hospital nurse; Chief Nursing Officer; Female; 59 years old]*

*“Yes, it is available. We offer Pap smear to our patients at the family planning center. There are nurses there on standby always, Monday to Friday, 8 am to 4 pm. So every woman can come in and have their Pap smear taken and the result will be out in no time. Just in 10 days, results will be out, so the facility is available” [Tertiary hospital doctor; Senior resident doctor; Male; 35 years old]*

*“In fact, we can do it even in the clinic that we are seeing them. We can screen them immediately, and we can even read the results immediately with VIA or put it on the slide, and send it to the histology lab. It’s easily accessible, but cost...hmmn... cost is the problem”. [Tertiary hospital doctor; Senior resident doctor; Male; 41 years old]*

*“I’ve said that we don’t have the test. There is no way we can do the screening here. So, they are being encouraged to go to the state level.” [PHC nurse; Nursing Officer; Female; 24 years old]*

#### Practice of cervical cancer screening.

Most of the health workers at the PHC do not do cervical cancer screening as a routine at their facilities; they refer the clients that present with suggestive symptoms to higher facilities. On a few occasions, visual inspection with acetic acid was done in PHCs, during organized outreaches The materials for VIA are not readily available in PHCs, which necessitates referral of patients. A head nurse from a PHC said she has never practised cervical cancer screening, although she has seen it done in the past.

*“About the cervical cancer screening, it is a good program. But in a local area, we don’t usually have the facility to carry out the cervical cancer screening. I could remember that there was a time that we have cancer screening outreach at Ajegunle during a former governor for breast and cancer of the cervix.” [PHC nurse; Principal Nursing Officer; Male; 48 years old]*

*“Okay, little experience I had about that, but I know that during cervical screening, there is this testing lotion that is being used, that the cervix will be infiltrated with it, and there could be discoloration of the cervix. There could be patches on the cervix, and the likes. Although I have not been able to set my hand on it to screen to do cervical screening, but I have seen it being done.” [PHC nurse; Assistant Chief Nursing Officer; Female; 46 years old]*

Some General Hospitals screen patients for cervical cancer, usually using VIA, while others don’t. In a particular General Hospital, VIA was done routinely for HIV positive women, via a programme by an international non-governmental organization. VIA is done in some general hospitals, but they often lack the reagents and have to refer the patients to tertiary institutions for Pap smear.

*“Most times, we send them for pap smear elsewhere, because it is not all the time we do have acetic acid to do VIA.” [Secondary hospital nurse; Chief Nursing Officer; Female; 51 years old]*

*“Some years back, we do it here. We were using acetic acid to check the cervix… But for years back, we could not get acetic acid. So we stopped doing the tests here.” [Secondary hospital nurse; Chief Nursing Officer; Female; 59 years old]*

*“I think I mentioned that earlier. The cervical cancer screening is mainly done at the heart-to-heart clinic. There is screening mainly for the people living with HIV, but not for the general population. There is no program for that at the moment.” [Secondary hospital doctor; Principal Medical Officer; Male; 44 years old]*

Health workers from both tertiary hospitals sampled attested that Pap smear test is carried out in their health facilities. In the tertiary hospitals, cervical cancer screening is done when patients present with symptoms of cervical cancer, and as an opportunistic screening for all women who present with gynaecological symptoms. Colposcopy is also done, when indicated. Screening is however, not the norm for women seeking maternal care.

*“We are aware that there are other types of screening, There is liquid based cytology, but we don’t, have it. We are a resource poor country, so we don’t do liquid based cytology. We cannot do HPV analysis, so we just do the conventional pap smear. Then for some women, we will go beyond Pap smear and we do colposcopy for them, because we have colposcopy machine…” [Tertiary hospital doctor; Senior resident doctor; Male; 35 years old]*

*“We do it routinely, especially for our infertility patients or patients with any gynaecological problem - It is a must.” [Secondary hospital nurse; Chief Nursing Officer; Female; 55 years old]*

Some health workers mentioned that they do just clinical inspection of the cervix without acetic acid or Lugol’s iodine, when women need to have an IUCD inserted as a form of screening, and refer women with suggestive lesions on inspection, for Pap smear.

*“We all know the gold standard is the Pap smear. But we don’t do the Pap smear here. What we do here basically is the ehmm, the clinical ehm, inspection examination using a speculum. That usually happens when you want to kind of ehm... When you are recommending IUCDs for women during family planning. Once the nurse sees some characteristics that are not normal, they call us the doctors. We examine, and ask for samples to be taken. Though the samples are not taken here. We have to refer them to centers where they will be taken. But that’s basically what we do here.” [Secondary hospital doctor; Senior Medical Officer; Female; 34 years old]*

#### Barriers to cervical cancer screening faced by health workers.

A major challenge for many health workers was the lack of equipment, consumables and training to conduct cervical cancer screening. Many said manpower shortage would make it difficult for them to add cervical cancer screening to their duties. It was mentioned that many patients were only interested in finding solutions to their presenting complaints and are less bothered about screening. This could be a barrier to health workers advising on screening. A health worker mentioned unconducive environment from large crowds and lack of privacy as a barrier to screening. Language was said to be a barrier by a few of the participants.

*“Erm it’s because we don’t have the facility and we don’t have the equipment on ground. So we still need to refer them. Then when we refer them, some will come back. In fact, the majority will not come back. Only a few will come back.” [Secondary hospital doctor; Principal Medical Officer; Male; 48 years old]*

*“Number one equipment, number two manpower. Because if we are many, we will train other people, most especially the skilled health workers; I mean the nurse. If I’m not around, you can do this. But manpower is the problem. We have no manpower. There is no equipment and all these things…No reagents, they are supposed to give us for the work...” [PHC nurse; Chief Nursing Officer; Female; 51 years old]*

*“Lack of training, either no availability of knowledge about that or inexperience.” [Secondary hospital nurse; Principal Nursing Officer; Female; 41 years old]*

*“The challenges we usually encounter is most time... Most times, when the patient gets to you... Most time it’s usually late… Somebody will tell you that my menses stopped five years ago, I just saw it from January, I’ve been seeing blood, I’ve been seeing blood. So, by the time you do vagina or speculum examination, you already see florid clinical signs that most times you really just need that Pap smear to confirm.” [Secondary hospital doctor; Senior Medical Officer; Male; 42 years old]*

*“Once patient asks about the cost of screening and you tell them it’s 15,000 naira,... They’ll tell you - Okay, doctor, I have heard’. And we lose them to follow-up hmmn. We don’t see them. Even if you call them on phone, they won’t pick up.” [Tertiary hospital doctor; Senior Resident Doctor; Male; 35 years old]*

#### Barriers to cervical cancer screening faced by patients.

The cost of the screening was a challenge mentioned by many of the participants. Long distance to referral centers with its attendant cost was also mentioned as a barrier. Some health workers said fear of the unknown was a challenge for some patients, as they were afraid of being diagnosed with cancer, hence their reluctance to be screened. Other challenges mentioned ranged from religious and cultural beliefs to a lack of interest, feeling embarrassed especially with a male health worker, fear of painful procedure, husband not giving consent, and poor awareness by patients.

*“Then financial… financial constraints… If they don’t have much money and they wish to do the test... You know they need to source for fund and all of that. Then religious beliefs... Then the husband too may not give consent too.” [Secondary hospital nurse; Principal Nursing Officer; Female; 36 years old]*

*“Okay, like the transportation problem? You know, from here to... Abeokuta is a bit far, so you have to...You have to...At least before you go there, you have to have some money with you to go there. And most of them, it is this money… financial problems… they complain about. So, it is transportation and the financial... And the distance, majorly...” [Secondary hospital nurse; Chief Nursing Officer; Female; 52 years old]*

*“Some can just feel ashamed and say; How can I just go and ask for a thing like that” [Secondary hospital nurse; Principal Nursing Officer; Female; 43 years old]*

*“Then, most of the time, it is lack of knowledge. Because they don’t really know the significance. Some of them have this disease and they think it is harmless… that it will go. So, it is basically knowledge based…”. [Secondary hospital doctor; Principal Medical Officer; Male; 47 years old]*

*“So, the barrier they may encounter is that - ‘If I enter and they said it’s positive, won’t that lead me to another thing?’ So that is the barrier people are facing. It’s better for me not to go, than to go there and hear, you are positive of this…” [PHC nurse; Assistant Chief Nursing Officer; Female; 44 years old]*

#### Facilitators of cervical cancer screening.

Patient’s complaint related to the sign and symptoms of cervical cancer, and passion for the job, were facilitators of cervical cancer screening mentioned. Cervical cancer screening as a routine procedure prior to provision of family planning was also mentioned as a facilitator.

*“We don’t want to lose our patients. We want them to be safe at all times.... Because we don’t want to lose them... Because we know the prevention is usually...it’s always better than cure.” [Secondary hospital nurse; Chief Nursing Officer; Female; 52 years old]*

*“Because I don’t have a choice. When they come for family planning, I have to check them. In some cervix you see erosion lesion that will even not allow you to insert IUCD. Usually, I don’t insert IUD when there is erosion. I rather send them for screening.” [Secondary hospital nurse; Chief Nursing Officer; Female; 51 years old]*

*“Like I said, most times, when the presenting complaint is related, so that encourages not only health education, then the screening itself. If the complaint is related, then we are motivated to screen.” [Secondary hospital doctor; Principal Medical Officer; Male; 44 years old]*

### Recommendations for cervical cancer health education and opportunistic screening

Recommendations for screening and health education by the health workers include; making cervical cancer screening and health education a routine at antenatal and postnatal clinics, appropriate remuneration and training of health workers, availability of equipment, reagents and screening centers, availability of adequate manpower and female staff, making screening free/more affordable/providing health insurance, organizing screening outreaches, creating awareness using radio, IEC materials and community mobilization/awareness, and allaying patients fears of screening.

*“If it’s actually incorporated as a routine… the counselling… into ANC. You won’t forget. You will know – I’ve not done this.” [Secondary hospital doctor; Senior Medical Officer; Female; 35 years old]*

*“And also there should be a kind of remuneration also, for health workers in order to do all of these things. Because when you’re being remunerated, apart from you having that eagerness or willing to do the job, but because there is remuneration, and you are being appreciated even if it’s not even about paying huge money - but be appreciated - Either by word or even in cash - you know, that will also help to facilitate and will help the health worker even to perform well”. [Secondary hospital nurse; Principal Nursing Officer; Female; 32 years old]*

*“So, if we have more centers...we have more screening centers. That’s where the government comes in. The government should be proactive about having centres in different local governments...” [Secondary hospital doctor; Principal Medical Officer; Male; 38 years old]*

*“Well, I think if we have the facilities, the equipment, the necessary things to get it done...so that, when you counsel them and they agree, you get them screened immediately. That will motivate them… And maybe even if a cost will be attached to it, it will be such that the cost will be so minimal that they can afford…. For example, if you said, okay, cervical cancer screening is just 500 naira or even not up to that, and the person is already aware of the benefits attached to it,…right in front of her, she will pay…” [PHC nurse; Assistant Chief Nursing Officer; Female; 44 years old]*

*“The health workers themselves should also be trained. Because what you don’t have, you cannot give.” [Secondary hospital doctor; Principal Medical Officer; Male; 53 years old]*

*“But assuming we have female personnel, they will feel free. At least they do come for delivery, and they feel free. Because they know nurses here - they are mostly female. We don’t even have male, so they are free. So, if the same thing applies to this screening, they may want to submit themself for the test.” [Secondary hospital nurse; Chief Nursing Officer; Female; 59 years old]*

*“There should be community-based awareness programme. Why I said this is that; many of the people in this environment will not come to the hospital to register. They prefer going to “alagbos”(traditional birth attendants) for their antenatal care. So, if you say until when they get to the hospital, you will give them information, what about those that are outside there? So public awareness, community awareness is important.” [Secondary hospital doctor; Principal Medical Officer; Male; 53 years old]*

*“Through giving them health information. And giving them free screening.” [Tertiary hospital nurse; Chief Nursing Officer; Female; 59 years old]*

*“I think cervical cancer screening should be made to be a part of the postnatal clinic because that’s where you have an exposure to larger number of women population” [Tertiary hospital doctor; Consultant Obstetrician & Gynaecologist; Male; 42 years old]*

## Discussion

This qualitative study sought to understand the practice of opportunistic cervical cancer screening and health education, and the barriers and facilitators experienced by health workers practicing in antenatal and postnatal clinics in Ogun state, Nigeria. All the health workers were aware of cervical cancer screening, even though there were knowledge gaps in risk factors knowledge and type of screening tests. Health education on cervical cancer was not practiced in some facilities and was generally less discussed compared with breast cancer screening. Cervical cancer health education was mostly practised by nurses during group health education at family planning clinics and sometimes at antenatal and immunization clinics. Most doctors only counselled on screening when their patients had suggestive symptoms. Supply-side challenges to cervical cancer screening and health education include facilities not being accessible, cost of screening, inadequate training of health workers, lack of reagents and equipment, manpower shortage and heavy workload. Demand-side challenges include fear of positive result among patients, fear of painful procedure, embarrassment, poor awareness and lack of interest in screening, religious and cultural beliefs, and husband not giving consent. The major facilitators to screening and health education mentioned by health workers were passion for their work and desire that no woman should die from preventable cancers. Pap smear was mainly available in the tertiary health facilities but is only routinely recommended to women with gynaecological complaints. Most PHCs lack facilities for screening, and only a few secondary health facilities have equipment/reagents for screening. Most times, screening was done only for patients with gynaecological complaints, and not for asymptomatic women/women seeking maternal healthcare.

### Health workers knowledge of cervical cancer screening

In this study, all the health workers were aware of cervical cancer screening. Similarly, in a study among female health workers in Sokoto, northern Nigeria, majority of the health workers knew about Pap smear as a cervical cancer screening method [24]. Also, among medical workers in Mulago hospital Uganda, most of the medical workers were aware of the Pap smear test [25].

Though awareness of cervical cancer screening was high in our study, some gaps in knowledge were noticed. Some health workers believed that screening was only for detecting cervical cancer, not for identifying pre-cancerous lesions in asymptomatic women. This misunderstanding could lead to missed opportunities for early intervention.

Also, some health workers equated just inspecting the cervix without the use of acetic acid or Lugol’s iodine to screening. Such beliefs by health workers could be deleterious, as this incorrect practice could lead to a large number of false negative cases. Our study also found that only a few health workers, mainly doctors working in tertiary health facilities, mentioned HPV testing as a screening method, despite this test, being the gold standard for cervical cancer screening. This puts forward the need for health workers to have up to date information on cervical cancer prevention, via continuing education. Similar to our study, a study among female healthcare professionals in Saudi Arabia, showed only a small proportion (10.6%) were aware of HPV DNA testing [26].

### General practice of health education in health facilities

Group health education was a common practice in most health facilities and was the routine in antenatal clinics. However, not all health facilities practice group health education during postnatal visits. In some cases, health education is given during family planning clinics and also in infant welfare clinics. Group health education during ANC covered several topics such as nutrition, care of the breast during pregnancy, immunization, personal and environmental hygiene, and importance of good health seeking behaviour. It is commendable that most facilities practice health education, as this forms a major part of disease prevention and health promotion. Group health education during antenatal visits is a common phenomenon across public hospitals in Africa, [20,21,27–29] This is a good practice, as every visit of a woman of childbearing age to the health facility for maternal or child health services should be utilized as an opportunity to provide important health information. This opportunity should also not be missed during postnatal clinics and family planning visits too.

### Practice of health education on cervical cancer prevention

When there was health education on cervical cancer prevention, the discussion, for many health workers focused mostly on avoidance of risk factors, such as multiple sexual partners, and did not really address cervical cancer screening. Such form of health education is incomplete and is not optimally beneficial to the patient. A combination of avoidance of risk factors and screening is needed for cervical cancer prevention. We also found that health workers more frequently discussed breast cancer screening compared to cervical cancer screening. This is partly explained by the higher incidence and perhaps, prevalence of breast cancer. What is worrisome is the disproportionate priority placed on cervical cancer, considering that it ranks 2^nd^ commonest cancer among women after breast cancer, in Nigeria. Breast cancer may also be more commonly discussed because care of the breast in preparation for breastfeeding is frequently discussed among pregnant women during health education, and in the process, health workers also talk about self-breast examination and breast cancer screening. Cervical cancer screening may however be viewed as not being not of immediate concern to the pregnant or nursing mother. Even though discussions around care of the breast are crucial in pregnancy, this period is a golden opportunity to educate women on cervical cancer screening also, as they may not have other opportunities to hear such important message.

Some health workers provide appropriate information to their patients on cervical cancer screening, by providing information on Pap smear and VIA. This is usually done by nurses. This practice should be encouraged. Among the doctors, most doctors only counsel their patients on screening when patients present with symptoms suggestive of cervical cancer, or other gynaecological symptoms. In a study carried out in a Ugandan tertiary hospital, among medical workers, who were predominantly nurses, majority of the medical workers, did not discuss cervical screening with their patients [25]. This may be because the health workers in the Ugandan study were drawn from different departments, compared to our study which was among health workers in antenatal and postnatal clinics.

### Misconceptions regarding cervical cancer prevention

Some health workers also had wrong knowledge of cervical cancer risk factors and this formed part of their health education, e.g., some health workers felt douching and using saliva as lubricant for sexual activity is a risk factor for cervical cancer. Even though such practices should be avoided, basing health education solely on such could have deleterious effects. Similarly, in a study among health workers in northern Malawi, [30] many health workers had poor knowledge of cervical cancer risk factors. However, in another study conducted among nurses, midwives, and clinical officers working in primary care in Uganda, most health workers had good knowledge of cervical cancer risk factors [31]. This may be because to achieve the targets of the Strategic Plan for Cervical Cancer Prevention and Control in Uganda, several implementation strategies which included training health workers in the primary health care system on cervical cancer prevention was done.

### Availability of cervical cancer screening services and practice of opportunistic screening

Regarding availability of cervical cancer screening services, Pap smear is available in the tertiary health facilities but is only routinely recommended to women with gynaecological complaints. A few secondary health facilities have facilities for screening while almost all the PHCs lack facilities for cervical cancer screening. Some PHCs had reagents for VIA in the past but not in recent times, and some had carried out outreaches in the past. Even though provision of maternal and child care, and treatment of endemic diseases is the major focus of primary health care, the government should also prioritize cervical cancer screening in PHCs and secondary health facilities, with provision of VIA at PHCs and VIA and Pap smear at secondary health facilities. It is also important to make HPV DNA testing, which is the gold standard for screening, available at tertiary health facilities.

In our study, many health workers only recommend cervical cancer screening to women that have gynaecological symptoms. Similarly, a qualitative study in Libya found that none of the health workers recommend cervical cancer screening to healthy women [26] It is recommended that health workers should offer screening to all women, and not just limit cervical cancer screening to women with suggestive symptoms. Health education on cervical cancer prevention can be provided during pregnancy, and opportunistic screening done at the 6^th^ week postnatal visit, as in being done in some tertiary facilities in Nigeria [32]. Our study found that in some health facilities, health workers only inspected the cervix of women, without applying acetic acid or Lugol’s iodine, usually during intrauterine contraceptive device insertion. Even though these health workers felt they were providing some form of cervical cancer screening, this practice could lead to many missed cases and also overdiagnosis. Proper screening with VIA, Pap smear or HPV testing should be practiced, and the government should ensure adequate provision of the required equipment and reagents.

### Barriers to cervical cancer screening and health education faced by health workers

Inadequate training was mentioned as a barrier to carrying out cervical cancer screening and health education by health workers. Lack of reagents and equipment, manpower shortage and heavy workload was also mentioned by health workers as barriers to screening and health education. In India [33] and Cameroon [34], inadequate infrastructure and trained human resource is also a barrier to cervical cancer control. Ministries of Health should ensure adequate training and re-training of staff on cervical cancer screening and health education. Adequate staffing is necessary for more effective provision of services in general, and also for provision of cervical cancer screening services. Language barrier was also an identified barrier to screening and health education, in some areas. This can be overcome by employing health workers who understand some of the minority languages. Community members who understand the minority language and English language can also serve as interpreters.

### Barriers to cervical cancer screening faced by patients

Some challenges patients face in accessing cervical cancer screening include facilities not being accessible, and cost of screening not being affordable. Many patients have to travel long distances to access cancer screening services. Also, the cost of Pap smear is beyond the easy reach of many. A qualitative study in Uganda [35] and another qualitative study in Nigeria, [36] also reported that cost of cervical cancer screening was a major barrier among women of low resource. Living far from screening centers was also documented as a barrier to screening by Adedimeji et al, in a study in Cameroon, where women had to travel long distances to reach cervical cancer screening centers [34]. It is pertinent for government to address issues of geographical and financial access to cancer screening services. For example, every LGA can have a basic cancer screening center, and screening can be made available free/at affordable cost/included in health insurance package.

Fear of positive result, fear of painful procedure, fear of being embarrassed, poor awareness and lack of interest in screening, religious and cultural beliefs, and husband not giving consent, were also mentioned by health workers as factors which may negatively impact screening. Many patients are afraid of the outcome of screening, and religious and cultural beliefs of chastity can discourage women from exposing themselves for screening. Religious beliefs that cancer cannot happen to them, can also prevent women from being screened. Similar barriers have been reported in other studies [35–41]. Hasahya et al. in Uganda reported fear of bad result and fear of the procedure as barriers to screening [41]. Nyamambi et al. in Zimbabwe [38] and Gupta et al. in India [33], reported cultural/spiritual beliefs as a barrier to screening. [38] Getachew et al. in Addis Ababa [39] and Ekawati in Indonesia [42] reported poor knowledge of cervical cancer as a barrier, and Adewumi in Kenya reported lack of spousal support [40]. Continuous health education on the benefits of screening, allaying fears, and advocacy to religious, traditional leaders and spouses, can help address these barriers. Health education and advocacy has been shown to improve cervical cancer screening uptake by involving women, traditional/religious leaders and spouses [43,44]. Health workers should not stop encouraging women to screen and should not be discouraged when some women refuse screening.

### Facilitators of cervical cancer screening and health education

Understanding facilitators, can help promote screening and health education, when these facilitators are further potentiated. The major facilitator of health workers to screening and health education were passion for their work and desire that no woman should die from preventable cancers. Other facilitators were patients’ interest and inquisitiveness to learn, positive response from the patients, e.g., carrying out the recommended screening tests, high educational status of the clients as they could easily comprehend the benefit of screening, and group health education being a routine practice. Even though a patient that is easy to work with, is a facilitator to health workers, health workers should not be dismayed when they encounter patients who may seem difficult to work with. In addition, many doctors said patient’s complaint related to the sign and symptoms of cervical cancer, would encourage them to screen such patient. However, screening should be offered to all women that meet the eligibility criteria irrespective of symptoms.

### Recommendations proffered by health workers for opportunistic cervical cancer screening

We asked health workers to proffer recommendations for opportunistic cervical cancer screening, after explaining what the term means to them. Recommendations proffered were: integrating cervical cancer education into routine health education sessions at antenatal and postnatal clinics, integrating cervical cancer screening into post-natal clinics, providing ongoing training and retraining for health workers to ensure they have up-to-date knowledge on cervical cancer screening methods, addressing supply-side barriers by increasing the availability of screening facilities, reagents, and equipment, making screening services more affordable or free, and consider including them in health insurance packages. Health workers also recommended providing renumeration for additional work, which can improve work morale, and ensuring adequate staffing levels to reduce workloads and allow for more focused health education efforts. To increase demand for cervical cancer screening, health workers recommended organizing community-based awareness programs, and to address cultural and religious barriers to screening, health workers recommended engaging religious and traditional leaders. Similar recommendations of making screening affordable, creating awareness and organising outreaches was made by women in a low resource community in Lagos, Nigeria [36]. Also, findings from a qualitative study in Ibadan, Nigeria showed primary care health workers and women wanted cervical cancer screening information to be include postnatal services [45].

### Strengths and weaknesses of the study

This study provides important information on the practice of cervical cancer opportunistic screening among health workers, the barriers faced and facilitators. This is one of the few studies that assessed these objectives among health workers in Nigeria. Most studies among health workers examine their knowledge, attitude and utilization of screening for themselves, rather than their practice of screening on patients [24,25,31,46–50]. This study thus adds considerably to the body of knowledge and provides significant information that can influence prevention efforts of cervical cancer in Nigeria. The qualitative nature of the study also helps elucidate on the practice of cervical cancer screening and health education, providing stories behind the prevailing circumstances. A limitation of this study is the possibility of health workers giving socially desirable responses (social desirability bias). This was minimized in this study by assuring participants of anonymity and that information gotten was not to apportion blame. Another limitation relates to asking demand-side (patient-relevant) questions from those in the supply-side of the health system (health workers). However, our findings on barriers to screening are similar to those from studies conducted among patients [51,36].

## Conclusion

This study reveals critical gaps in cervical cancer screening and education within the studied health facilities. Despite widespread awareness, health workers demonstrated limited knowledge of risk factors and screening methods, with education efforts overshadowed by those for breast cancer. This, coupled with limited access to screening, cost barriers, and inadequate manpower and facilities for screening contributes to a reactive rather than proactive approach to cervical cancer prevention, primarily focused on symptomatic women. To address these deficiencies, it is vital for the government to invest in training programs for healthcare providers, recruit sufficient personnel, and ensure adequate supply of screening reagents, consumables and equipment. Screening should be made more affordable or free. Expanding grassroots awareness campaigns can help inform communities and improve access to screening. Embedding cervical cancer education and screening into standard maternal health services, such as antenatal and postnatal visits, is a key strategy for boosting participation rates.

## Supporting information

S1 Text In-depth interview topic guide. (PDF)
