# Supplementary material for: Practice of opportunistic cervical cancer screening and health education among health workers in Ogun State, Nigeria: A qualitative study of barriers and facilitators
Source: PLoS One. 2025 Sep 11;20(9):e0316883. doi: 10.1371/journal.pone.0316883 (PMC12425320; doi:10.1371/journal.pone.0316883)
Supplement: S1 Text — (PDF) [file pone.0316883.s001.pdf]

IN-DEPTH INTERVIEW GUIDE

1. Can you tell me what you know about breast cancer screening?
2. Can you tell me what you know about cervical cancer screening?
3. How is your practice of health education in this facility?
   a. Do you have health talks during the antenatal visits?
   b. Do you have health talks during the postnatal visits? Do you have separate postnatal clinic days where you attend to mothers and neonates? Please expatiate
   c. What are the topics frequently discussed during health education at antenatal/postnatal clinics?
   d. In your health facility, do you have discussions about breast cancer prevention during health education? Please expatiate.
   e. In your health facility, do you have discussions about cervical cancer prevention during health education? Please expatiate.
4. Please tell me about your practice of health education/counselling personally, as a health worker
   a. As a doctor/nurse, do you counsel your patients on breast cancer prevention during group sessions/one on one encounters (probe: in ANC/PNC clinic and other clinics)? Please expatiate
   b. As a doctor/nurse, do you counsel your patients on cervical cancer prevention e.g. need for screening, during group sessions/one on one encounters (probe: in ANC/PNC clinic and other clinics)?
   c. How frequently do you counsel your patients on breast and cervical cancer prevention in ANC/PNC clinic .
5. Do you usually recommend breast cancer screening for your patients (probe: in ANC/PNC and other clinics)? Please expatiate.
   a. What form of breast cancer screening, Under what conditions, How frequent?
6. Do you usually recommend cervical cancer screening for your patients (probe: in ANC/PNC and other clinics)? Please expatiate.
   b. What form of cervical cancer screening, Under what conditions, How frequent?
7. Are facilities for breast cancer screening easily accessible to your patients? Please expatiate.
8. Are facilities for cervical cancer screening easily accessible to your patients? Please expatiate.

9. What do you think are the barriers/challenges, patients face in accessing breast cancer screening? Please expatiate
10. What do you think are the barriers/challenges, patients face in accessing cervical cancer screening? Please expatiate
11. What barriers/challenges do you experience in offering breast cancer screening to your patients? Please expatiate
12. What barriers/challenges do you experience in offering cervical cancer screening to your patients? Please expatiate
13. What barriers/challenges do you experience in offering breast and cervical cancer health education to your patients?
14. In your current practice, what are the things or conditions that encourage you to offer breast and cervical cancer health education to your patients?
15. In your current practice, what are the things or conditions that encourage you to offer breast cancer screening to your patients (probe: during ANC/PNC and other clinics)?
16. In your current practice, what are the things or conditions that encourage you to offer cervical cancer screening to your patients (probe: during ANC/PNC and other clinics)?

Briefly explain: Opportunistic screening is when we use the opportunity that women come for ANC or PNC to screen them for cervical cancer and breast cancer. It is important because ANC and PNC is a place many women will pass through in their life experience, and this could be a good opportunity to educate women about cancer, screen them or refer them for screening.

17. What do you think can be done to promote breast cancer screening for pregnant women/mothers during ANC/PNC in your facility.
18. What do you think can be done to promote cervical cancer screening for pregnant women/mothers during ANC/PNC in your facility.
19. What do you think can be done to promote breast cancer health education for pregnant women/mothers in your facility.
20. What do you think can be done to promote cervical health education for pregnant women/mothers in your facility.
